# Association of Peripheral Arterial Occlusive Disease and Deep Venous Thrombosis with Risk of Consequent Sepsis Event: A Retrospective Population-Based Cohort Study

**DOI:** 10.3390/ijerph19116710

**Published:** 2022-05-31

**Authors:** Bo-Yuan Wang, Ying-Hsiang Chou, Chi-Tzu Chung, Shun-Fa Yang, Shu-Ling Tzeng, Yu-Hsun Wang, Ming-Chih Chou, Chao-Bin Yeh, Chi-Ho Chan

**Affiliations:** 1 Institute of Medicine, Chung Shan Medical University, Taichung 402, Taiwan; chibochichiyasu@gmail.com (B.-Y.W.); p1234737785@gmail.com (C.-T.C.); ysf@csmu.edu.tw (S.-F.Y.); cherie@csmu.edu.tw (S.-L.T.); cshy236@csh.org.tw (M.-C.C.); sky5ff@gmail.com (C.-B.Y.); 2 Department of Emergency Medicine, School of Medicine, Chung Shan Medical University, Taichung 402, Taiwan; 3 Department of Emergency Medicine, Chung Shan Medical University Hospital, Taichung 402, Taiwan; 4 Department of Radiation Oncology, Chung Shan Medical University Hospital, Taichung 402, Taiwan; hideka.chou@gmail.com; 5 Department of Medical Imaging and Radiological Sciences, Chung Shan Medical University, Taichung 402, Taiwan; 6 Department of Medical Research, Chung Shan Medical University Hospital, Taichung 402, Taiwan; cshe731@csh.org.tw; 7 Department of Surgery, Chung Shan Medical University Hospital, Taichung 402, Taiwan; 8 Department of Microbiology and Immunology, Chung Shan Medical University, Taichung 402, Taiwan

**Keywords:** peripheral arterial occlusive disease, deep vein thrombosis, sepsis, risk

## Abstract

Peripheral artery occlusive disease (PAOD) and deep vein thrombosis (DVT) can cause a variety of acute and chronic vascular complications and put patients at risk of subsequent sepsis. This study aimed to determine whether DVT compared with PAOD patients would increase the risk of sepsis. This study recruited 43,535 patients newly diagnosed as having PAOD and 6932 patients who were newly diagnosed as having DVT from a population of 2 million patients from the Longitudinal Health Insurance Database. Propensity score matching (PSM) between the PAOD and DVT groups was performed for age, sex, comorbidities, and prior antibiotic administration. A total of 4383 patients with PAOD and 4383 patients with DVT were analyzed for risk of sepsis. The incidence density of sepsis per 1000 person years for patients with PAOD was 25.75 (95% CI = 23.90 to 27.74) and 35.61 (95% CI = 33.29 to 38.09) for patients with DVT. After age, sex, associated comorbidities, and antibiotic administration were adjusted for, the risk of sepsis for the DVT group was 1.46-fold (95% CI = 1.32–1.62) higher than that for the PAOD group. In conclusion, patients with DVT were associated with a higher risk of subsequent sepsis than patients with PAOD. Aging was another risk factor.

## 1. Introduction

Peripheral arterial occlusive disease (PAOD) is a clinical presentation of systemic atherosclerosis that is necessarily associated with coronary artery disease, carotid artery disease, and cerebrovascular diseases [1]. The main causes of PAOD are the formation of thrombosis or embolism in the arteries of the lower limbs, and PAOD can result in severe atherosclerotic stenosis, leading to loss of limb or even death [2]. The incidence of PAOD varies by country. In the United States and Taiwan, 14 and 0.6 people out of 100,000 people in the general population experience PAOD, respectively [2,3]. This indicates that the incidence of PAOD in Taiwan was lower than that of the USA. Although many patients with PAOD experience intermittent claudication, some are asymptomatic. The known risk factors of PAOD include smoking, diabetes mellitus, hypertension, and hyperlipidemia [4].

Deep venous thrombosis (DVT) is the formation of a blood clot (thrombus) in a deep vein and causes phlebitis. DVT can develop into a pulmonary embolism when untreated. Similar to PAOD, DVT can occur in various other areas, such as upper and lower limbs, visceral veins, etc., with over 90% of all DVTs located in the lower extremity [5], but the causes of DVT are different from those of PAOD. The most probable pathogenesis of DVT is Virchow’s triad, consisting of blood flow stasis, endothelial damage, and increased blood viscosity [6,7]. A meta-analysis including nine studies, which were conducted in Sweden or the USA mostly, estimated that the weighted mean incidence of DVT in the general population was approximately 5.04 per 10,000 per year [8]. However, little information exists about the incidence of DVT in Taiwan [9], although 16–17 cases of venous thromboembolism (VTE) per 100,000 person years among the general population have been reported [7]. Many diseases have been associated with DVT. For example, congestive heart disease, liver cirrhosis, excessive abdominal pressure, chronic venous insufficiency, and clotting factor deficiencies. In addition, patients with several types of malignancies, such as thyroid cancer, multiple myeloma, or lung cancer are also at an increased risk for DVT [10]. Furthermore, patients with primary valvular reflux and prolonged orthostatic position, which leads to venostasis, may be considered as the high-risk group of DVT [11,12].

Sepsis is a nonspecific systemic illness that is caused by overwhelming microbial infection, especially by Gram-negative bacteria [13]. Severe sepsis can cause multiple organ dysfunction and the occurrence of a cytokine storm. The mechanism of organ dysfunction is a complete process, including cellular dysoxia, interorgan interactions, and alterations in cellular metabolism [14]. Patients with sepsis may experience septic shock and death [13,15]. The incidence and mortality rates in Taiwan for any severity level of sepsis were 643 and 287 per 100,000 people, respectively, and the average case fatality rate was 29.2% from 2010 through 2014 [16].

Many studies have indicated that both PAOD and DVT exhibit endothelial damage, dysfunction, and long-term inflammation of blood vessels [6,17,18,19,20]. Therefore, patients with PAOD or DVT being more susceptible to developing sepsis during infection was a reasonable assumption. However, few studies have examined the association between PAOD and DVT with sepsis.

In this study, we enrolled patients with PAOD or DVT from the Longitudinal Health Insurance Database 2000 (LHID 2000) to elucidate the association among sepsis. We hypothesized that DVT compared with PAOD patients would increase the risk of sepsis.

## 2. Materials and Methods

### 2.1. Study Design and Participants

The LHID is regulated by the Health and Welfare Data Science Center in Taiwan. The database contains data on all outpatient and inpatient medical claims, including diagnosis of disease with the international classification of diseases—Ninth Revision (ICD-9), medications, surgical operations, procedures, and fees from 2000 through 2015. The database covers 2 million beneficiaries that were randomly sampled by stratification according to age, sex, and region from the entire population of the 2000 registry for beneficiaries in Taiwan. By linking the 2000 registry for beneficiaries and Longitudinal Health Insurance Database, we can trace the patient’s medical record from 2000 through 2015. The study was approved by the Ethical Review Board of the Chung Shan Medical University Hospital (CS1-20056).

### 2.2. Study Group and Outcome

This study had a retrospective cohort study design. The two-study population comprised patients aged ≥20 years, with 43,535 who were newly diagnosed as having PAOD (ICD-9-CM codes: 443.8, 443.9, and 444) or 6932 DVT (ICD-9-CM codes = 453.8) from 2001 to 2014. To ensure accurate diagnosis, outpatient visits ≥ 2 times or hospitalizations ≥ 1 time were stipulated. The index date was the date of the first diagnosis of PAOD or deep vein thrombosis during admission. To reduce the confounding of coexisting disease in each group, there were 41,624 and 4844 patients in this study after excluding patients with diagnoses of DVT and PAOD from the PAOD and DVT groups, respectively. A total of 3068 and 499 patients with a diagnosis of sepsis (ICD-9-CM codes: 038, 995.91, and 995.92) before the index date were excluded; we did so to include only patients with new-onset PAOD or DVT. The outcome variable was a diagnosis of sepsis on hospitalization or a visit to the emergency department. Both groups were followed-up until the onset of sepsis, death, or 31 December 2015, whichever occurred first.

### 2.3. Covariates and Matching

The baseline characteristics were age, sex, hypertension (ICD-9-CM codes: 401 to 405), hyperlipidemia (ICD-9-CM codes: 272.0 to 272.4), diabetes (ICD-9-CM code: 250), ischemic heart disease (ICD-9-CM codes: 410 to 414), chronic kidney disease (ICD-9-CM code: 585), chronic obstruction pulmonary disease (ICD-9-CM codes: 491, 492, and 496), intracranial bleeding (ICD-9-CM codes: 430 to 432), stroke (ICD-9-CM codes: 433 to 438), malignancy (ICD-9-CM codes: 140 to 208), rheumatoid arthritis (ICD-9-CM code: 714.0), systemic lupus erythematosus (SLE; ICD-9-CM code: 710.0), Sjogren’s syndrome (ICD-9-CM code: 710.2), ankylosing spondylitis, (ICD-9-CM code: 720.0), and psoriasis (ICD-9-CM codes: 696.0 and 696.1). In our analysis, a patient was considered to have any one of these comorbidities if they had a diagnosis of the comorbidity within 1 year prior to the index date and either made at least two outpatient visits or were admitted at least once for the comorbidity in question. In addition, data on antibiotic use within 1 year prior to the index date and for ≥4 courses were included for analysis.

We conducted 1:1 propensity score matching (PSM) between the two groups for age, sex, hypertension, hyperlipidemia, diabetes, ischemic heart disease, chronic kidney disease, chronic obstruction pulmonary disease, intracranial bleeding, stroke, malignancy, rheumatoid arthritis, systemic lupus erythematosus, Sjogren’s syndrome, ankylosing spondylitis, and psoriasis, prior antibiotic use, and index year. The propensity score was estimated through logistic regression. The binary variable was a presence or absence in either the DVT or the PAOD group. By matching the propensity score, it could reduce the heterogeneity and had a similar starting time among both groups. At last, there were 4383 patients in each group.

### 2.4. Statistical Analysis

The DVT and PAOD groups were compared using the absolute standardized difference, where a value < 0.1 was defined as indicating similarity between both groups [21]. The relative risk (RR) and 95% confidence intervals (CI) were calculated using Poisson regression. Kaplan–Meier analysis was used to calculate the cumulative incidence of sepsis among the two groups. A log-rank test was used to test for significance. To determine the independent risk in the DVT group compared with PAOD group, a multivariate Cox proportional hazard model was used to estimate the hazard ratios. Statistical analysis was conducted in SAS version 9.4 (SAS Institute, Cary, NC, USA).

## 3. Results

### 3.1. Characteristics of the Participants

**Figure 1:**
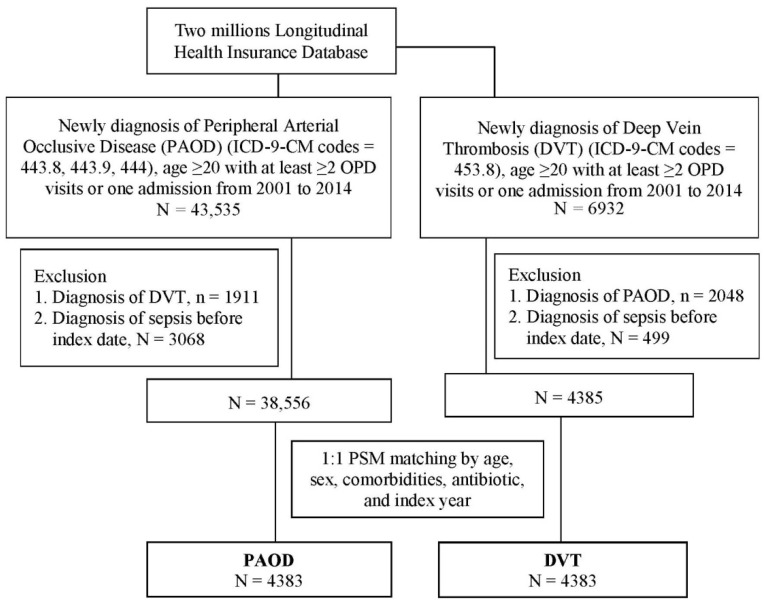
Flowchart of patient selection.

In total, 43,535 and 6932 patients who were newly diagnosed as having PAOD and DVT, respectively, were recruited from the 2,000,000 patients in the LHID. After the patients with DVT were excluded from the PAOD group and vice versa, and after patients who were diagnosed with sepsis before the index date were excluded, 1:1 PSM was conducted between patients with PAOD and patients with DVT by age, sex, comorbidities, antibiotic use, and index year. Finally, 4383 patients with PAOD and the same number of patients with DVT with similar age, sex, comorbidity, antibiotic use, and index year characteristics were analyzed for their risk of sepsis (Figure 1). A comparison of the characteristics of the PAOD and DVT patients is presented in Table 1.

### 3.2. Risk of Sepsis between DVT and PAOD Group

**Figure 2:**
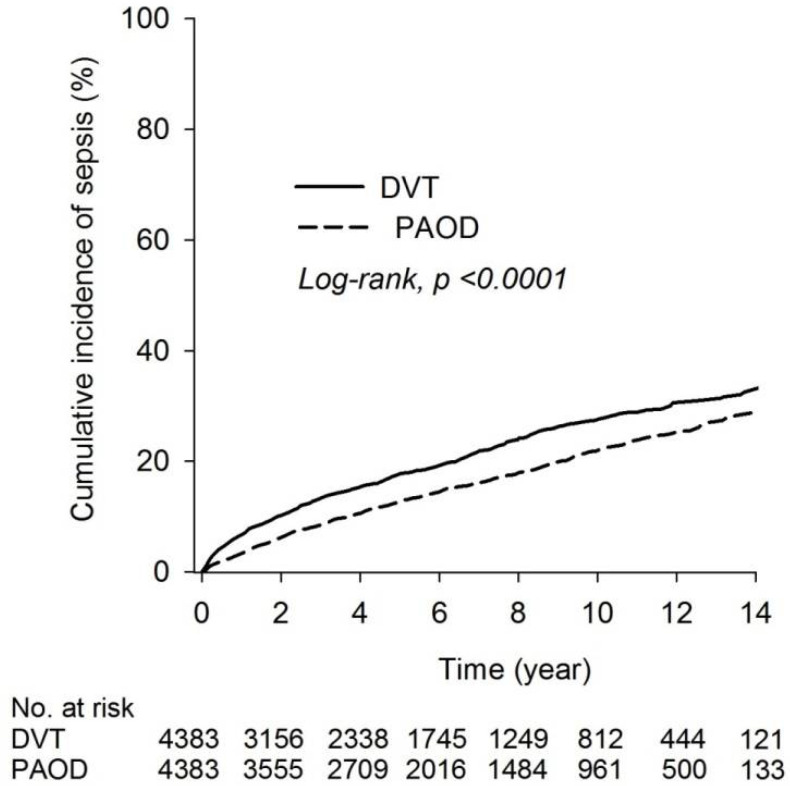
Kaplan–Meier curves of the cumulative proportions of sepsis in DVT and PAOD patients.

The incidence densities of sepsis were 25.75 (95% CI = 23.90 to 27.74) and 35.61 (95% CI = 33.29 to 38.09) patients per 1000 person years in the PAOD and DVT groups, respectively. The RR of sepsis was 1.38-fold (95% CI = 1.25 to 1.53) higher in the DVT group than in the PAOD group (Table 2). The cumulative incidence of sepsis risk in both groups revealed that the risk of sepsis was higher in the DVT group than in the PAOD group (log-rank test, *p* < 0001; Figure 2).

### 3.3. Comparison of Risk of Sepsis between DVT and PAOD Group

After age, sex, associated comorbidities, and antibiotic use were adjusted for, the risk of sepsis in the DVT group was 1.42-fold (95% CI = 1.29 to 1.58) higher than that in the PAOD group. In addition, diabetes, chronic kidney disease, chronic obstructive pulmonary disease, intracranial bleeding, stroke, malignancy, and SLE were risk factors for sepsis. By contrast, patients who received antibiotics 1 year before their index date still had an increased risk of sepsis (HR = 1.36; 95% CI = 1.22 to 1.51; Table 3). Moreover, the risk of mortality in the DVT group was 1.54-fold (95% CI = 1.44 to 1.66) higher than that in the PAOD group after adjusting age, sex, associated comorbidities, and antibiotic use (Supplemental Table S1). Furthermore, the chemotherapy uses and transplant organ status are shown in Supplemental Table S2. The data showed the DVT group had higher chemotherapy use. However, after controlling chemotherapy and transplant organ status, the DVT group had significant higher sepsis risk than the PAOD group (HR = 1.41; 95% CI = 1.27 to 1.56) (Supplemental Table S3).

### 3.4. Subgroup Analysis of Sepsis Risk in DVT Group Relative to PAOD Group after PSM

In a subgroup analysis, patients in the DVT group aged 20 to 39 years (HR = 2.28; 95% CI = 1.25 to 4.16), aged 40 to 64 years (HR = 1.50; 95% CI = 1.22 to 1.83), or aged ≥65 years (HR = 1.34; 95% CI = 1.19 to 1.51) had a higher risk of sepsis relative to patients in the PAOD group. Additionally, in the DVT group, women (HR = 1.43; 95% CI = 1.24 to 1.63), men (HR = 1.45; 95% CI = 1.24 to 1.69), and both patients with a history of antibiotic treatment (HR = 1.39; 95% CI = 1.18 to 1.65) or no antibiotic treatment (HR = 1.44; 95% CI = 1.27 to 1.63) showed a risk of sepsis (Table 4).

## 4. Discussion

Patients newly diagnosed with PAOD or DVT were enrolled to analyze their association with sepsis. Our results indicated that the risk of sepsis was found to be higher in the DVT group than in the PAOD group. Patients with DVT exhibited a higher risk of sepsis than patients with PAOD. While PAOD can develop necrosis, ulcer, and gangrene in final stage, patients with DVT might develop complications such as pulmonary embolism, post-thrombotic syndrome (PTS), and phlegmasia cerulea dolens (PCD) [22,23]. Patients with PTS would appear venous ulceration, while patients with serious PCD would lead to gangrene. We found that these complications might be associated with sepsis and explain the reasons why DVT patients are easier to have sepsis than PAOD patients. We reviewed studies that have examined the pathologic changes to the endothelium in patients with DVT or PAOD. We found that endothelial damage in the blood vessels can alter the endothelium and cause endothelial dysfunction in both DVT and PAOD.

Endothelial damage and dysfunction in PAOD have been reported. An elevated level of homocysteine derived from oral methionine intake was also a predisposing factor for atherosclerosis through damage to the vascular tissue and was the cause of endothelial dysfunction in patients with PAOD [17]. Tsakiris’s research group compared the level of various soluble cell adhesion molecules (CAMs) in patients with PAOD before and after receiving percutaneous transluminal angioplasty. These CAMs included P-selectins and E-selectins, ICAM-1, and VCAM-1. Other markers, such as thrombomodulin, von Willebrand factor (vWF), and homocysteine, that reflect endothelial injury have also been detected. Their results indicated that a significant proportion of patients with PAOD had increased levels of these CAMs and endothelial markers in blood. This indicated that the endothelium of the arteries was in an active state in these patients [24]. Certain inflammatory factors, such as CRP, fibrinogen, interleukin-6 (IL-6), and matrix metalloproteinases were also found in the blood in patients with PAOD [19]. Studies on CAMs and PAOD were summarized in Brevett’s publication [25]. By contrast, plasma levels of adiponectin were lower in patients with PAOD [18]. Because adiponectin acts as a regulator of fat metabolism and is also associated with the attenuation of the inflammatory response in the endothelium, lower levels of adiponectin may contribute to more serious inflammation.

Several studies examined the endothelial damage and dysfunction in DVT. A literature review indicated that, in patients in the acute phase of VTE, endothelial cells produced IL-6, platelet-activating factor, and other substances that affect the inflammatory response and involve the formation of thrombi [26]. Persistently increased levels of inflammatory factors, such as tumor necrosis factor alpha (TNF-α), IL-6, and CXCL-8, accompanied by certain markers of endothelial damage, such as tissue plasminogen activator (tPA), plasminogen activator inhibitor 1 (PAI-1), and vWF, were detected in patients with a history of idiopathic DVT. These findings indicate that DVT causes long-term inflammation [6]. Recently, the term “immunothrombosis” has been used to refer to the pathogenesis of VTE involving the innate immune response. Thus, thrombus formation involves both coagulation problems and their associated immune reactions and inflammation processes [20].

The aforementioned findings jointly indicate that (1) PAOD and DVT aggravate the inflammatory response in endothelial cells and cause endothelial dysfunction, and (2) patients with PAOD and DVT have a higher risk of sepsis after infection. Hereditary factors related to the activity of the endothelium may also affect the risk of sepsis in human beings. Endothelial protein C receptor (EPCR), a receptor expressed on the endothelium of large blood vessels and correlated to the suppression of proinflammatory cytokine synthesis, may influence the risk of sepsis [27]. However, genetic polymorphism exists in the EPCR gene. For instance, people with different alleles on the gene may be at a higher risk of sepsis during infection [28].

Age is a risk factor for sepsis. In recent studies, sepsis was reported to be more prevalent among older patients, especially patients aged >85 years [29,30]. Additionally, aging was associated with delayed time of recovery from sepsis after treatment [31]. However, age may not be the only factor. Lifestyle-related factors and comorbidities in older adult patients may increase the risk of sepsis [32]. Our study reported aging was associated with higher sepsis risk.

The difference in susceptibility to sepsis between men and women has been examined. A systematic review of 12 multicenter randomized controlled trials reported a greater prevalence of sepsis in men than in women [33]. This difference was strongly associated with the level of sex hormones [34]. The higher levels of cytokines (i.e., IL-6, IL-10, and CXCL-8) released by men may explain why men exhibit more serious and systemic inflammatory responses [35]. Consequently, men had a higher hospital mortality rate than did women for severe sepsis among patients > 50 years old. By contrast, even women < 50 years old with multiple organ dysfunction syndromes had a lower mortality rate than men did [36,37]. Another reason is that female sex hormones may be associated with improved major organ function, especially in women of reproductive age [38]. In our study, we also observed that men had a higher risk of acquiring sepsis than woman did, and our result is consistent with those of the studies mentioned herein.

A previous study indicated that administration of antibiotics could improve outcomes in patients with septic shock [39]. In subgroup analysis of risk of sepsis, we also want to examine whether patients with a history of antibiotic treatment would affect the risk of sepsis in patients. However, our result indicated that history of antibiotic treatment could not lower the risk of sepsis on both PAOD and DVT patients in our study. One of the reasons might be, if the antibiotic use was inappropriate, the outcome might increase the development of multidrug-resistant bacteria [40].

The characteristics that are shared by PAOD and DVT have seldom been researched. Wuillemin et al. observed that high homocysteine plasma levels were associated with atherosclerotic, vascular, and venous thromboembolic diseases [41]. Plasma levels of homocysteine above the 95th percentile were found to accompany a twofold to threefold increased RR for DVT, even for individuals with mild hyperhomocysteinemia, which has been associated with a twofold to fourfold elevated related risk for PAOD. However, several studies and clinical reports have reported associations among bacterial infections, PAOD, and DVT. Chiu et al. conducted a retrospective cohort study by using 2000 to 2010 data from the Taiwan NHIRD [42]. Patients with leptospirosis exhibited higher risks of PAOD than did the general population. By contrast, in an epidemiologic case–control study conducted between October 1990 and December 1991, Samama et al. reported that several infectious diseases were risk factors for developing DVT. Moreover, an observational cohort study in Denmark for the 1995 to 2008 period reported that the risk of developing DVT increased within the first year after the onset of *Staphylococcus aureus* bacteremia [43]. Furthermore, Smeeth et al. reported that the risk of DVT increased after urinary tract and respiratory tract infection [44]. Tichelaar et al. reported that both inflammatory diseases and infections were risk factors for the development of DVT [45]. In summary, infectious diseases are associated with PAOD and DVT.

This study has some limitations. First, the database did not provide information about the severity of sepsis or the duration of antibiotic administration. These factors may affect sepsis risk. Second, the database did not provide information about the types of pathogens that caused sepsis in patients, which is relevant because sepsis virulence varies between microbes. Third, data on health behaviors, such as physical activity and diet, were not obtained from the database. Fourth, this study’s retrospective cohort design precludes causal inference. Fifth, due to propensity score matching, reduction in the PAOD group would contain selection bias.

## 5. Conclusions

In this study, patients with DVT were associated a higher risk of subsequent sepsis than patients with PAOD. Further medical researchers were needed to clarify the mechanisms between DVT and PAOD.

## Figures and Tables

**Table 1:** Demographic characteristics of PAOD and DVT.

	Before PSM Matching			After PSM Matching		
	PAOD (*N* = 38,556)	DVT (*N* = 4385)	ASD	PAOD (*N* = 4383)	DVT (*N* = 4383)	ASD
Age, Mean ± SD	63.31 ± 14.72	62.36 ± 16.31	0.061	62.65 ± 15.77	62.36 ± 16.31	0.018
Female sex	20,194 (52.4)	2617 (59.7)	0.148	2596 (59.2)	2615 (59.7)	0.009
Hypertension	18,823 (48.8)	1845 (42.1)	0.136	1893 (43.2)	1845 (42.1)	0.022
Hyperlipidemia	7899 (20.5)	629 (14.3)	0.163	610 (13.9)	629 (14.4)	0.012
Diabetes	10,483 (27.2)	764 (17.4)	0.236	750 (17.1)	764 (17.4)	0.008
Ischemic heart disease	6892 (17.9)	667 (15.2)	0.072	679 (15.5)	667 (15.2)	0.008
Chronic kidney disease	2038 (5.3)	326 (7.4)	0.088	308 (7.0)	326 (7.4)	0.016
COPD	2936 (7.6)	322 (7.3)	0.010	335 (7.6)	322 (7.4)	0.011
Intracranial bleeding	307 (0.8)	62 (1.4)	0.059	52 (1.2)	61 (1.4)	0.018
Stroke	4801 (12.5)	431 (9.8)	0.083	423 (9.7)	431 (9.8)	0.006
Malignancy	1666 (4.3)	679 (15.5)	0.380	699 (16.0)	677 (15.5)	0.014
Rheumatoid Arthritis	468 (1.2)	52 (1.2)	0.003	39 (0.9)	52 (1.2)	0.029
SLE	76 (0.2)	31 (0.7)	0.076	17 (0.4)	30 (0.7)	0.041
Sjogren’s syndrome	292 (0.8)	31 (0.7)	0.006	18 (0.4)	31 (0.7)	0.040
Ankylosing spondylitis	83 (0.2)	16 (0.4)	0.028	14 (0.3)	16 (0.4)	0.008
Psoriasis	144 (0.4)	21 (0.5)	0.016	10 (0.2)	21 (0.5)	0.042
Antibiotic	9837 (25.5)	1260 (28.7)	0.072	1238 (28.3)	1259 (28.7)	0.011

DVT: deep vein thrombosis; ASD: absolute standardized differences; COPD: chronic obstruction pulmonary disease; SLE: systemic lupus erythematosus; PAOD: peripheral arterial occlusion disease; PSM: propensity score matching; SD: standard deviation.

**Table 2:** Poisson regression of relative risk of PAOD and DVT.

	Before PSM		After PSM	
	PAOD	DVT	PAOD	DVT
*N*	38,556	4385	4383	4383
Person-years	229,482.07	23,772.84	26,954	23,756
No. of sepsis	5859	846	694	846
ID (95% C.I.)	25.53 (24.88–26.19)	35.59 (33.27–38.07)	25.75 (23.90–27.74)	35.61 (33.29–38.09)
Relative risk (95% C.I.)	Reference	1.39 (1.30–1.50)	Reference	1.38 (1.25–1.53)

ID: incidence density (per 1000 person years); DVT: deep vein thrombosis; CI: confidence interval; PAOD: peripheral arterial occlusion disease; PSM: propensity score matching.

**Table 3:** Cox proportional hazard model analysis for risk of sepsis.

	Univariate		Multivariate †	
	HR (95% C.I.)	*p* Value	HR (95% C.I.)	*p* Value
Group				
PAOD	Reference		Reference	
DVT	1.37 (1.24–1.51)	<0.001	1.42 (1.29–1.58)	<0.001
Age	1.06 (1.05–1.06)	<0.001	1.05 (1.04–1.05)	<0.001
Sex				
Female	Reference		Reference	
Male	1.39 (1.26–1.54)	<0.001	1.11 (1.00–1.23)	0.059
Hypertension	2.05 (1.85–2.26)	<0.001	0.96 (0.86–1.08)	0.511
Hyperlipidemia	1.12 (0.97–1.30)	0.116	-	-
Diabetes	2.25 (2.01–2.53)	<0.001	1.54 (1.37–1.74)	<0.001
Ischemic heart disease	1.76 (1.56–1.99)	<0.001	1.02 (0.90–1.16)	0.759
Chronic kidney disease	2.89 (2.50–3.34)	<0.001	2.42 (2.09–2.80)	<0.001
COPD	2.56 (2.22–2.96)	<0.001	1.46 (1.26–1.70)	<0.001
Intracranial bleeding	2.80 (2.07–3.77)	<0.001	1.88 (1.38–2.55)	<0.001
Stroke	2.70 (2.37–3.07)	<0.001	1.58 (1.38–1.82)	<0.001
Malignancy	2.12 (1.87–2.41)	<0.001	1.84 (1.62–2.09)	<0.001
Rheumatoid Arthritis	1.18 (0.74–1.88)	0.481	-	-
SLE	2.16 (1.30–3.59)	0.003	4.18 (2.50–6.99)	<0.001
Sjogren’s syndrome	1.11 (0.56–2.23)	0.762	-	-
Ankylosing spondylitis	0.42 (0.10–1.66)	0.215	-	-
Psoriasis	1.37 (0.65–2.87)	0.411	-	-
Antibiotic	1.59 (1.43–1.76)	<0.001	1.38 (1.24–1.53)	<0.001

HR: hazard ratio; C.I.: confidence interval; DVT: deep vein thrombosis; COPD: chronic obstruction pulmonary disease; SLE: systemic lupus erythematosus; PAOD: peripheral arterial occlusion disease. † Adjusted for age, sex, hypertension, diabetes, ischemic heart disease, chronic kidney disease, COPD, intracranial bleeding, stroke, malignancy, SLE, and antibiotic.

**Table 4:** Subgroup analysis of risk of sepsis.

	Peripheral Artery Occlusive Disease (PAOD)		Deep Venous Thrombosis (DVT)			
	*N*	No. of Sepsis	*N*	No. of Sepsis	HR (95% C.I.)	*p* Value
Age ^1^						
20–39	423	16	447	38	2.28 (1.25–4.16)	0.007
40–64	1790	164	1868	231	1.50 (1.22–1.83)	<0.001
≥65	2170	514	2068	577	1.34 (1.19–1.51)	<0.001
p for interaction = 0.2031						
Sex ^2^						
Female	2596	371	2615	488	1.43 (1.24–1.63)	<0.001
Male	1787	323	1768	358	1.45 (1.24–1.69)	<0.001
p for interaction = 0.9210						
Antibiotic ^2^						
No	3145	432	3124	549	1.44 (1.27–1.63)	<0.001
Yes	1238	262	1259	297	1.39 (1.18–1.65)	<0.001
*p* for interaction = 0.5823						

^1.^ Adjusted for age, sex, hypertension, hyperlipidemia, diabetes, ischemic heart disease, chronic kidney disease, intracranial bleeding, stroke, malignancy, and antibiotic. ^2.^ Adjusted for age, sex, hypertension, hyperlipidemia, diabetes, ischemic heart disease, chronic kidney disease, intracranial bleeding, stroke, malignancy, rheumatoid arthritis, Sjogren’s syndrome, ankylosing spondylitis, psoriasis, and antibiotic. HR: Hazard Ratio.

## Data Availability

Restrictions apply to the availability of these data. Data were obtained from National Health Insurance database and are available from the authors with the permission of National Health Insurance Administration of Taiwan.

## Supplementary Materials

The following supporting information can be downloaded at: https://www.mdpi.com/article/10.3390/ijerph19116710/s1. Supplemental Table S1: Cox proportional hazard model analysis for risk of mortality. Supplemental Table S2: Chemotherapy use and transplant organ status among PAOD and DVT group. Supplemental Table S3: The risk of sepsis among DVT compared with PAOD group.
