# Supplementary material for: Association of Peripheral Arterial Occlusive Disease and Deep Venous Thrombosis with Risk of Consequent Sepsis Event: A Retrospective Population-Based Cohort Study
Source: Int J Environ Res Public Health. 2022 May 31;19(11):6710. doi: 10.3390/ijerph19116710 (PMC9180137; doi:10.3390/ijerph19116710)
Supplement: Supplementary file 1 [file ijerph-19-06710-s001.zip › ijerph-1626697-supplementary.pdf]

Supplemental Table S1. Cox proportional hazard model analysis for risk of mortality

	Univariate		Multivariate †	
	HR (95% C.I.)	p value	HR (95% C.I.)	p value
Group				
PAOD	Reference		Reference	
DVT	1.40 (1.30-1.50)	<0.001	1.54 (1.44-1.66)	<0.001
Age	1.06 (1.06-1.07)	<0.001	1.06 (1.05-1.06)	<0.001
Sex				
Female	Reference		Reference	
Male	1.80 (1.68-1.93)	<0.001	1.43 (1.33-1.54)	<0.001
Hypertension	1.90 (1.77-2.04)	<0.001	0.89 (0.82-0.96)	0.004
Hyperlipidemia	0.91 (0.82-1.02)	0.110	-	-
Diabetes	2.07 (1.91-2.25)	<0.001	1.49 (1.37-1.62)	<0.001
Ischemic heart disease	1.73 (1.59-1.88)	<0.001	1.01 (0.92-1.10)	0.834
Chronic kidney disease	2.61 (2.35-2.90)	<0.001	2.24 (2.02-2.49)	<0.001
COPD	2.45 (2.22-2.72)	<0.001	1.34 (1.20-1.49)	<0.001
Intracranial bleeding	1.74 (1.35-2.24)	<0.001	1.24 (0.96-1.60)	0.104
Stroke	2.11 (1.92-2.33)	<0.001	1.23 (1.11-1.36)	<0.001
Malignancy	3.09 (2.85-3.35)	<0.001	2.67 (2.46-2.90)	<0.001
Rheumatoid Arthritis	1.21 (0.87-1.67)	0.254	-	-
SLE	1.22 (0.77-1.93)	0.407	-	-
Sjogren's syndrome	1.05 (0.63-1.74)	0.859	-	-
AS	0.97 (0.50-1.86)	0.919	-	-
Psoriasis	1.31 (0.78-2.22)	0.311	-	-
Antibiotic	1.45 (1.35-1.57)	<0.001	1.20 (1.11-1.29)	<0.001

† Adjusted for age, sex, hypertension, diabetes, ischemic heart disease, chronic kidney disease, COPD, intracranial bleeding, stroke, malignancy, SLE, and antibiotic.

Supplemental Table S2. chemotherapy use and transplant organ status among PAOD and DVT group.

	PAOD (N = 4383)	DVT (N = 4383)	ASD
Chemotherapy	160 (3.7)	292 (6.7)	0.137
Transplant organ status	20 (0.5)	31 (0.7)	0.033

Supplemental Table S3. The risk of sepsis among DVT compared with PAOD group

	Univariate		Multivariate†	
	HR (95% C.I.)	p value	HR (95% C.I.)	p value
Group				
PAOD	Reference		Reference	
DVT	1.37 (1.24-1.51)	<0.001	1.41 (1.27-1.56)	<0.001
Age	1.06 (1.05-1.06)	<0.001	1.05 (1.05-1.05)	<0.001
Sex				
Female	Reference		Reference	
Male	1.39 (1.26-1.54)	<0.001	1.10 (0.99-1.21)	0.087
Hypertension	2.05 (1.85-2.26)	<0.001	0.97 (0.87-1.09)	0.626
Hyperlipidemia	1.12 (0.97-1.30)	0.116	-	-
Diabetes	2.25 (2.01-2.53)	<0.001	1.54 (1.37-1.74)	<0.001
Ischemic heart disease	1.76 (1.56-1.99)	<0.001	1.00 (0.88-1.14)	0.969
Chronic kidney disease	2.89 (2.50-3.34)	<0.001	2.43 (2.10-2.81)	<0.001
COPD	2.56 (2.22-2.96)	<0.001	1.48 (1.28-1.73)	<0.001
Intracranial bleeding	2.80 (2.07-3.77)	<0.001	1.90 (1.40-2.58)	<0.001
Stroke	2.70 (2.37-3.07)	<0.001	1.60 (1.40-1.84)	<0.001
Malignancy	2.12 (1.87-2.41)	<0.001	1.68 (1.48-1.92)	<0.001
Rheumatoid Arthritis	1.18 (0.74-1.88)	0.481	-	-
SLE	2.16 (1.30-3.59)	0.003	4.28 (2.56-7.16)	<0.001
Sjogren's syndrome	1.11 (0.56-2.23)	0.762	-	-
AS	0.42 (0.10-1.66)	0.215	-	-
Psoriasis	1.37 (0.65-2.87)	0.411	-	-
Antibiotic	1.59 (1.43-1.76)	<0.001	1.37 (1.23-1.52)	<0.001
Chemotherapy	2.03 (1.68-2.46)	<0.001	1.90 (1.56-2.32)	<0.001
Transplant organ status	1.04 (0.56-1.93)	0.905	-	-

⧫ Adjusted for age, sex, hypertension, diabetes, ischemic heart disease, chronic kidney disease, COPD, intracranial bleeding, stroke, malignancy, rheumatoid arthritis, SLE, antibiotic, and chemotherapy.
